# Multi-Trial Gait Adaptation of Healthy Individuals during Visual Kinematic Perturbations

**DOI:** 10.3389/fnhum.2017.00320

**Published:** 2017-06-20

**Authors:** Trieu Phat Luu, Yongtian He, Sho Nakagome, Kevin Nathan, Samuel Brown, Jeffrey Gorges, Jose L. Contreras-Vidal

**Affiliations:** Noninvasive Brain-Machine Interface System Laboratory, Department of Electrical and Computer Engineering, University of HoustonHouston, TX, United States

**Keywords:** locomotor adaptation, visuo-motor adaptation, gait symmetry, human locomotion, motor adaptation

## Abstract

Optimizing rehabilitation strategies requires understanding the effects of contextual cues on adaptation learning. Prior studies have examined these effects on the specificity of split-belt walking adaptation, showing that contextual visual cues can be manipulated to modulate the magnitude, transfer, and washout of split-belt-induced learning in humans. Specifically, manipulating the availability of vision during training or testing phases of learning resulted in differences in adaptive mechanisms for temporal and spatial features of walking. However, multi-trial locomotor training has been rarely explored when using visual kinematic gait perturbations. In this study, we investigated multi-trial locomotor adaptation in ten healthy individuals while applying visual kinematic perturbations. Subjects were instructed to control a moving cursor, which represented the position of their heel, to follow a prescribed heel path profile displayed on a monitor. The perturbations were introduced by scaling all of the lower limb joint angles by a factor of 0.7 (i.e., a gain change), resulting in visual feedback errors between subjects' heel trajectories and the prescribed path profiles. Our findings suggest that, with practice, the subjects learned, albeit with different strategies, to reduce the tracking errors and showed faster response time in later trials. Moreover, the gait symmetry indices, in both the spatial and temporal domains, changed significantly during gait adaptation (*P* < 0.001). After-effects were present in the temporal gait symmetry index whens the visual perturbations were removed in the post-exposure period (*P* < 0.001), suggesting adaptation learning. These findings may have implications for developing novel gait rehabilitation interventions.

## New and noteworthy

This study deployed a novel gait adaptation paradigm in which healthy subjects learned to move their lower limbs to control a cursor in response to visuo-motor perturbations (i.e., gain change). Our findings suggest that, with practice, the subjects learned, albeit with different strategies, to reduce the tracking errors and showed faster response time in later trials. Moreover, we observed differential effects of adaptation to the kinematic perturbation on the temporal and spatial symmetry (i.e., after-effects were only observed in the temporal domain of gait symmetry, indicating its adaptation). Overall, these findings may have implications for the development of novel gait interventions for people with lower-limb disabilities.

## Introduction

Motor adaptation can be defined as an error-driven process that allows humans to adjust sensorimotor mappings of well-learned movements to adapt to new, predictable demands (Bastian, 2008; Malone et al., 2011). Thus, motor adaptation mechanisms are generally engaged in response to changing intrinsic (e.g., muscle fatigue, aging, and neurological disease) and/or extrinsic (e.g., visual kinematic or dynamic perturbations) conditions. Motor adaptation to extrinsic perturbations has been investigated in the context of gait rehabilitation for people with walking disabilities (Torres-Oviedo et al., 2011). Both visual and dynamic disturbances induce sensorimotor errors, which initiate motor adaptation. Indeed, experimental studies of motor adaptation have been performed by applying extrinsic perturbations including introducing abnormal visual feedback (Contreras-Vidal and Kerick, 2004; Krakauer et al., 2005; Cheng and Sabes, 2007; Wei and Kording, 2009; Izawa and Shadmehr, 2011; Luu et al., 2015, 2016), manipulating physical dynamics (split-belt treadmill; Scheidt et al., 2001; Choi and Bastian, 2007; Emken et al., 2007; Izawa et al., 2008; Malone et al., 2012), or both (Kim et al., 2010).

Helm and Reisman have reviewed the split-belt walking paradigm, which has been widely used to explore motor learning and spatiotemporal asymmetry for post-stroke conditions (Helm and Reisman, 2015). The locomotor adaptation to split-belt treadmill training can improve walking symmetry in post-stroke patients (Reisman et al., 2007), and the persistence of improved gait symmetry on a treadmill partially transfers to over-ground walking (Reisman et al., 2009). Moreover, the temporal and spatial control for symmetric gait can be adapted separately, which suggests we could potentially develop interventions targeting either temporal or spatial domain of gait deficits (Malone et al., 2012). However, split-belt treadmill is not the only way to disrupt normal gait patterns. In some cases, this purpose can also be achieved by altering the visual-motor representation of locomotion.

Prior studies have examined the effect of altering visual feedback in walking adaptation. Torres-Oviedo and Bastian showed that removing vision via blindfolds improved the transfer of split-belt treadmill adaptation to natural walking (Torres-Oviedo and Bastian, 2010). This agreed with a prior study that reported the transferring of motor learning occurs in the intrinsic coordinates (Imamizu et al., 1998; Malfait et al., 2002). Prisms are used in many studies to distort vision and interrupt the visuo-motor neural pathway (Morton and Bastian, 2004; Alexander et al., 2013; Nemanich and Earhart, 2015). On the other hand, visual cues can be provided and manipulated in rehabilitation. Kim et al. used a cursor on a screen to represent the sagittal position of a subject's foot when walking with a robotic system (Kim et al., 2010). They suggested that combining both dynamics (interaction force between subjects and robot) and visual perturbations to the gait pattern retained the gait adaptation for longer than training with either dynamics or visual perturbation alone. Statton et al. used bars on a screen to show subjects the amount of their knee flexion (Statton et al., 2016). By manipulating the height of the bars, they created illusions in subjects that they had to over-correct their gait. Long et al. showed the position of steps on a screen so that the subjects would know where their feet landed (Long et al., 2016). Although the effect induced in the gait pattern from these studies vary greatly, they can usually be labeled as either spatial (Long et al., 2016; Statton et al., 2016) or temporal (Hussain et al., 2013; Finley et al., 2014) gait patterns but not both, partially limited by the paradigm of interruption to the visual-motor system.

In this study, we proposed a novel method to explore multi-day gait adaptation of human treadmill walking under visual kinematic perturbations. Participants were instructed to control a moving cursor, representing the heel position in the sagittal plane, to follow a specific trajectory displayed on a screen. Visual kinematic perturbations were introduced by scaling the lower limb joint angles (factor of 0.7) therefore distorting the mapping of the actual heel position on the screen, resulting in mismatch between the moving cursor and the prescribed heel path. Participants adapted their locomotor patterns to visual perturbations as demonstrated by a reduction of tracking errors across trials. After-effects were present when the visual perturbations were removed in the post-exposure period suggesting adaptation learning had taken place. We also found that individual subjects are idiosyncratic in which joints they use to control the heel positions during the adaptation. The modifications in the spatial and temporal symmetry of gait induced by the visual kinematic perturbations were also investigated. These findings have implications for developing novel effective gait rehabilitation interventions.

## Methods

### Experimental setup and procedure

Ten healthy individuals (six males, four females; aged from 22 to 30) with no history of neurological disease or lower limb pathology participated in this study. All participants provided written and informed consent as approved by the Institutional Review Board at the University of Houston. Each subject participated in two sessions (two consecutive days, two trials per session). There were two phases in each day at trial 1 and trial 3 (Table 1). In the first phase, the subjects were instructed to walk normally and consistently for 3 min on a treadmill at a fixed speed of 1 mile per hour (mph), or 0.45 meter per second (m/s). A 52-inch TV monitor was placed in front of the treadmill at eye level and it showed a black screen in this phase. The subjects were also instructed to look at the screen during the entire protocol. Heel path profiles of their normal walking were computed for each session from the goniometer data using Matlab 2016a (The MathWorks, Inc.). Details of the calculation are provided in Section Forward Kinematic Model. In the second phase, the subjects first stood still (rest) on the treadmill for 2 min, then started walking at 1 mph without visual feedback on the screen for 5 min, following by a walking period with visual feedback. The subjects stood still for another 2 min at the end of each trial. The walking period without the visual feedback serves as a baseline to normalize gait symmetry indices. The visual feedback to the subjects included a moving cursor, which was linked to their right heel position in the sagittal plane, and a desired heel path. During the entire visual feedback period, the subjects were instructed to control a moving cursor to follow prescribed right heel path profiles displayed on the screen. The tracking task was mainly spatial because the subjects performed the task without considering the time to reach a specific point. In the pre-exposure phase (8 min) in Trial 1, the moving cursor was driven by the goniometer data (right hip, knee, and ankle). In the exposure period (8 min), a visual kinematic perturbation was introduced by scaling the goniometer data of the right leg by 0.7 and the moving cursor departed from the prescribed heel profiles. The subjects controlled the moving cursor to follow the prescribed heel profiles by adapting their gait patterns. A gain of 0.7 was chosen because the visual perturbation is detectable with this gain and the physical demand to compete the task at this gain is not so high. In the post-exposure period (5 min), the perturbations were removed and the moving cursor was driven by the goniometer data again. We defined early- and late- exposure phases in each trial as the first and the last 30 gait cycles (~1 min), respectively. Similarly, early post-exposure phase was defined as the first 30 gait cycles in the post-exposure period. For safety purposes, all subjects were instructed to hold onto a front handle bar while walking on the treadmill.

**Table 1:** The experimental procedure for 4 trials in 2 consecutive days.

**Phase 1**			**Phase 2**					
**Template generation**			**Rest**	**Without visual feedback**	**With visual feedback**			**Rest**
					**Pre-**	**Exposure**	**Post-**	
Day 1	Trial 1	3	2	5	8	8	−	2
	Trial 2	−	2	5	−	8	−	2
Day 2	Trial 3	3	2	5	−	8	−	2
	Trial 4	−	2	5	−	8	5	2

*Heel path profiles (template generation) were generated in Phase 1. In Phase 2, subjects stood for 2 min in the beginning and the end (rest) and walked in periods without and with visual feedback. Small dash “−” represents periods that were excluded in a trial. All values are in minutes*.

### Data collection

**Figure 1:**
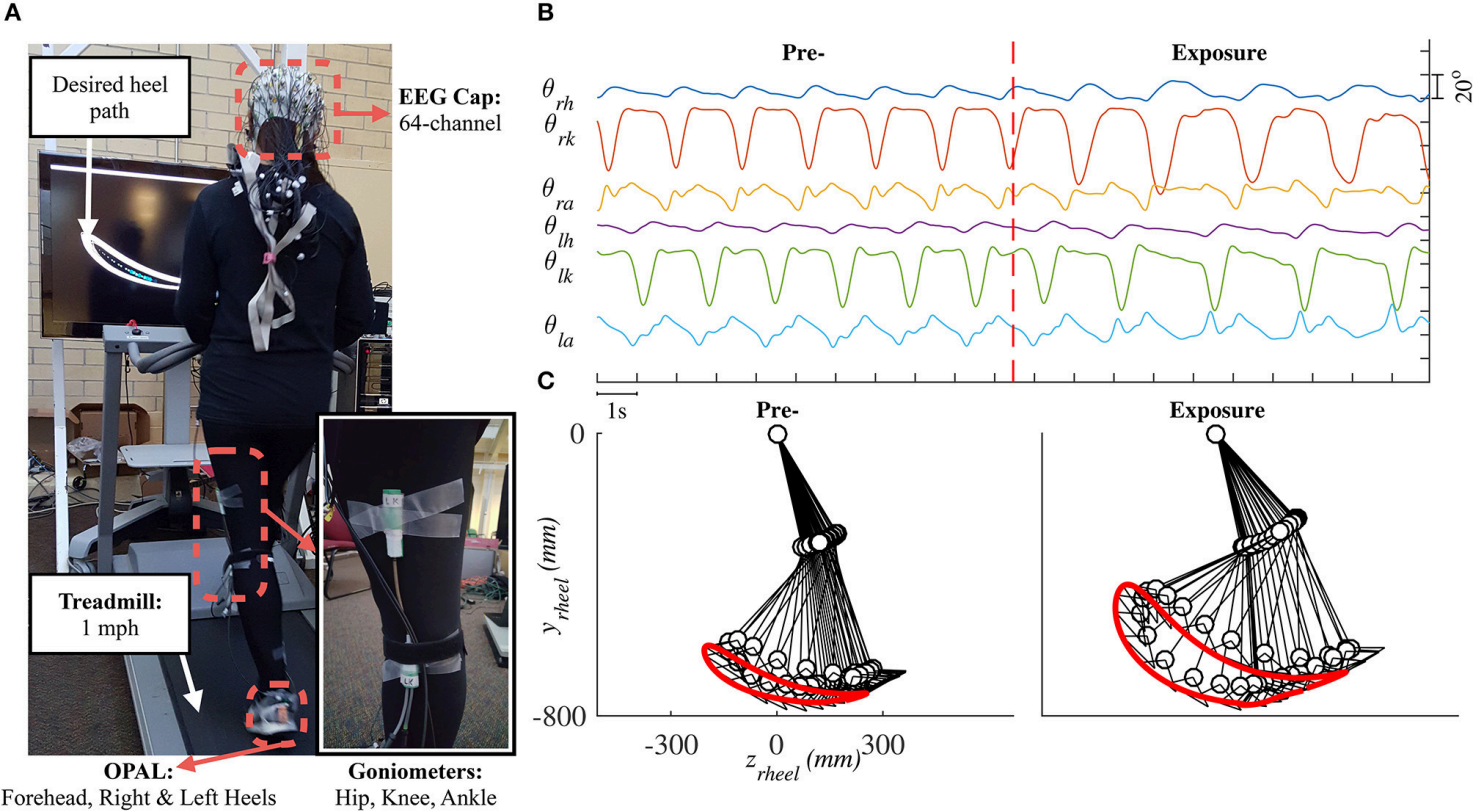
**(A)** Experimental setup in this study. Each subject was instructed to walk on a treadmill while controlling a moving cursor to follow prescribed heel path profiles. Lower limb joint angles (θ_*h*_, θ_*k*_, θ_*a*_) were recorded using six goniometers. **(B)** Example of joint angles in 20 s, vertical red line represents the time when the perturbation was introduced. **(C)** Illustration of the right leg in a full gait cycle during pre- and early-exposure.

Lower limb joint angles (hip, knee, and ankle) in the sagittal plane were recorded by goniometers (SG150 & SG110/A Gonio electrodes, Biometrics Ltd., UK) at 100 Hz using our customized C++ program. We visually estimated the joint locations and placed six sensors on both legs. To improve the consistency of sensor placement across days, we used the same sensor for each joint, 3D-printed a tool to hold the two pieces of a goniometer in place, and recorded the distance (from the ground) of each goniometer after the sensors were attached. Details for the goniometer sensor setup are provided in the Supplementary Material. Three wireless inertial motion sensors (OPAL, APDM Inc., Portland, OR) were mounted on the head, left heel, and right heel of the subject. Each sensor included accelerometer, gyroscope, and magnetometer sampled at 128 Hz. Electroencephalography (EEG) signals were also collected but will be reported elsewhere. Figure 1 shows raster plot of these signals and illustrates the movements of the right leg in a full gait cycle during pre- and early-exposure phases.

### Data analysis

Data analysis and statistical analysis were performed using custom software written in Matlab R2016a (The MathWorks, Inc.). Joint angle data were low-pass filtered at 6 Hz by using a second order Butterworth filter. Because subjects walked at a slow speed (1 mph), the 0–6 Hz band covers most power of joint angle signals (Antonsson and Mann, 1985; Luu et al., 2014). Heel position in sagittal plane was calculated using a forward kinematic model (Section Forward Kinematic Model).

### Forward kinematic model

**Figure 2:**
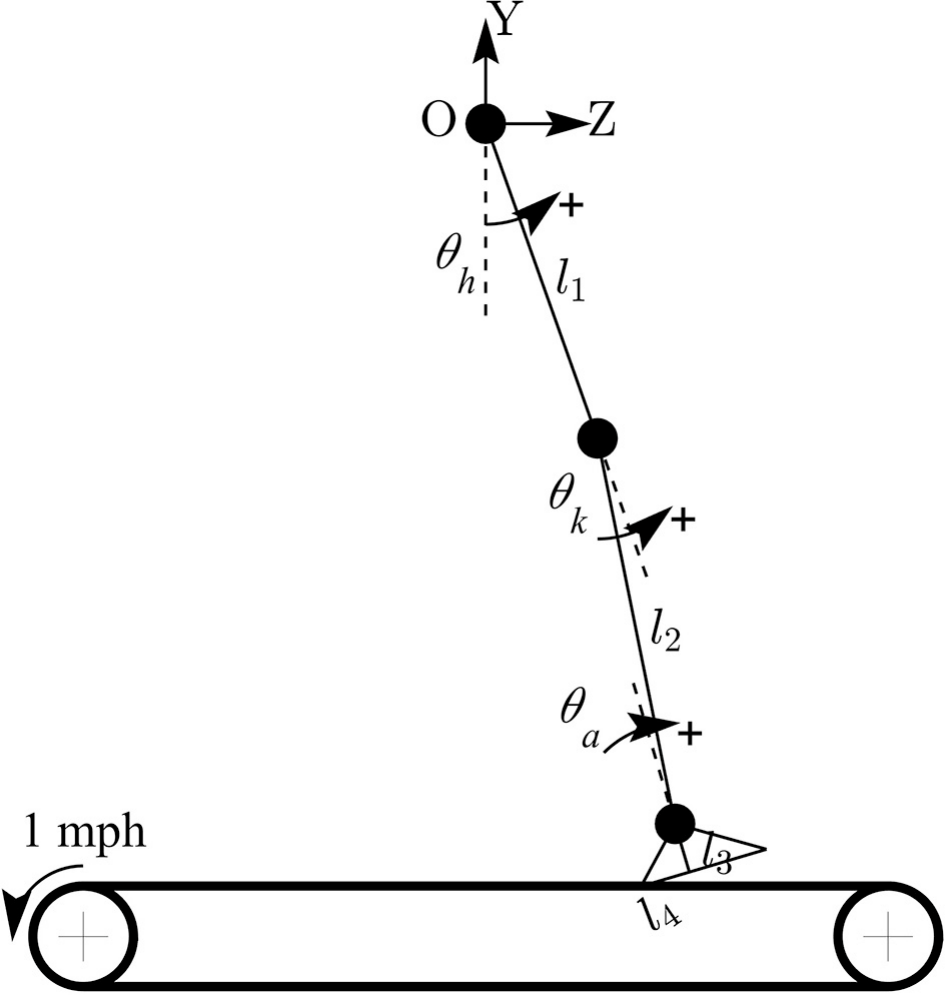
Forward kinematic model of human walking in sagittal plane. Coordinate OZY was placed at hip position and hip joint position is assumed to be fixed. Hip joint angle was defined to increase during flexion, knee joint angle increased during extension, and ankle joint angle increased during plantar-flexion.

The forward kinematic model of human treadmill walking (in sagittal plane) and the definitions of lower limb joint angles are shown in Figure 2. In this model, the hip joint position is assumed to be fixed. The prescribed heelpaths and trajectories of moving circle displayed on a screen during experiment were calculated by using the following equations:

(1) $$\begin{array}{l} z_{heel}=l_{1}\sin(\theta_{h})+l_{2}\sin(\theta_{h}+\theta_{k})+l_{3}\sin(\theta_{h}+\theta_{k}+\theta_{a})\\ \;+l_{4}\cos(\theta_{h}+\theta_{k}+\theta_{a})\\ y_{heel}=-l_{1}\cos(\theta_{h})-l_{2}\cos(\theta_{h}+\theta_{k})-l_{3}\cos(\theta_{h}+\theta_{k}\\ \;+\theta_{a})+l_{4}\sin(\theta_{h}+\theta_{k}+\theta_{a}) \end{array}$$

where *z*_*heel*_ and *y*_*heel*_ are horizontal and vertical heel position in sagittal plane, respectively; θ_*h*_, θ_*k*_, θ_*a*_ are hip, knee, and ankle joint angles, respectively; *l*_1_ is the length from the greater trochanter to the lateral epicondyle of the femur, *l*_2_ is the length from the lateral epicondyle of the femur to the lateral malleolus of the fibula, *l*_3_ is the perpendicular distance from the lateral malleolus of the fibula to the plantar surface of the foot, and *l*_4_ is the distance from the posterior aspect of the calcaneus (heel) to the projection of ankle onto the foot.

To assess the rate of change of *y*_*heel*_ with respect to the changes of each lower limb joint angle, the gradient of *y*_*heel*_ was also derived as:

(2) $$\begin{array}{l} \nabla y_{heel}=\left[\begin{array}{c} \frac{\partial y_{heel}}{\partial \theta_{h}}\\ \frac{\partial y_{heel}}{\partial \theta_{k}}\\ \frac{\partial y_{heel}}{\partial \theta_{a}} \end{array}\right]\\ \;=\left[\begin{array}{l} l_{1}\sin\left(\theta_{h}\right)+l_{2}\sin\left(\theta_{h}+\theta_{k}\right)+l_{3}\sin\left(\theta_{h}+\theta_{k}+\theta_{a}\right)\\ \;+\;l_{4}\cos\left(\theta_{h}+\theta_{k}+\theta_{a}\right)\\ l_{2}\sin\left(\theta_{h}+\theta_{k}\right)+l_{3}\sin\left(\theta_{h}+\theta_{k}+\theta_{a}\right)\\ \;+\;l_{4}\cos\left(\theta_{h}+\theta_{k}+\theta_{a}\right)\\ l_{3}\sin\left(\theta_{h}+\theta_{k}+\theta_{a}\right)\;+\;l_{4}\cos\left(\theta_{h}+\theta_{k}+\theta_{a}\right) \end{array}\right] \end{array}$$

### Gait segmentation and temporal gait parameters

**Figure 3:**
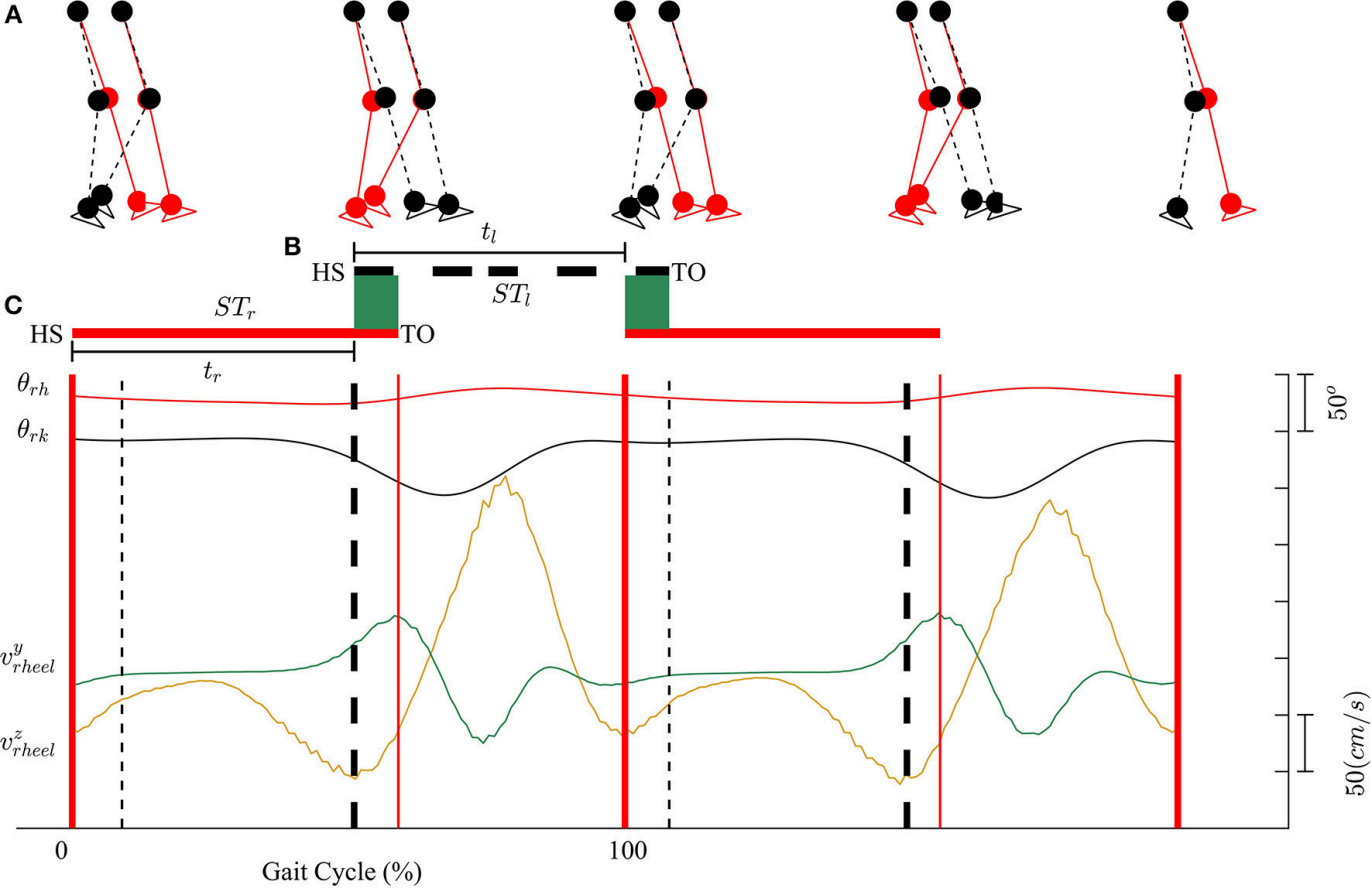
**(A)** Diagram for human walking at different gait events, walking speed 1 mph. **(B)** Definitions of temporal parameters of human locomotion. HS, heel strike; TO, toe-off. Solid and dashed horizontal lines represent stance time period (*ST*) for the right (*ST*_*r*_) and left (*ST*_*l*_) legs, respectively. Shaded areas indicate double support periods when both feet are on the ground; *DS*_*r*_ and *DS*_*l*_ are the right and left double support periods, respectively. Step times (interlimb heel-strike durations) of the right and left legs (*t*_*r*_ and *t*_*l*_) are defined as the time between consecutive heel-strikes. **(C)** θ_*rh*_, and θ_*rk*_ are hip and knee joint angles of the right leg, respectively. *v*_*yheel*_, and *v*_*zheel*_ are velocity profiles of heel in y and z directions. Thick solid and dashed vertical lines represent gait events for the right and left legs, respectively.

Kinematic data were segmented into gait cycles. Data from each trial were first divided into non-overlapping windows (window size of 10 s). Heel-strike and toe-off events for right and left legs were determined by using heel velocity profiles in the sagittal plane. Toe-off events in each window were defined by local maximum peaks of the heel velocity profile in the vertical direction (*v*_*yheel*_) (Winter, 1992). The minimum distance between two consecutive peaks is 80% of signal period which was computed by using auto-correlation method. Subsequently, heel-strike was determined by the first local minimum peak of heel velocity profile in the horizontal direction (*v*_*zheel*_) between two consecutive toe-offs. Figure 3 illustrates the alignment of gait events with lower limb joint angles and heel velocity profiles. Definitions of temporal gait parameters are also depicted. *ST* is stance time period from heel-strike (*HS*) to toe-off (*TO*) for the same leg. Double support time *DS* in a walking gait cycle is the time when both feet are in contact with the floor. Right double support, *DS*_*r*_, was defined as the time from left heel-strike to right toe-off, and vice versa for left double support, *DS*_*l*_. Interlimb heel-strike duration (*t*) is defined as the time between the heel-strike of one leg to the subsequent heel-strike of the other.

### Assessment of subjects' performance during visual kinematic perturbations

**Figure 4:**
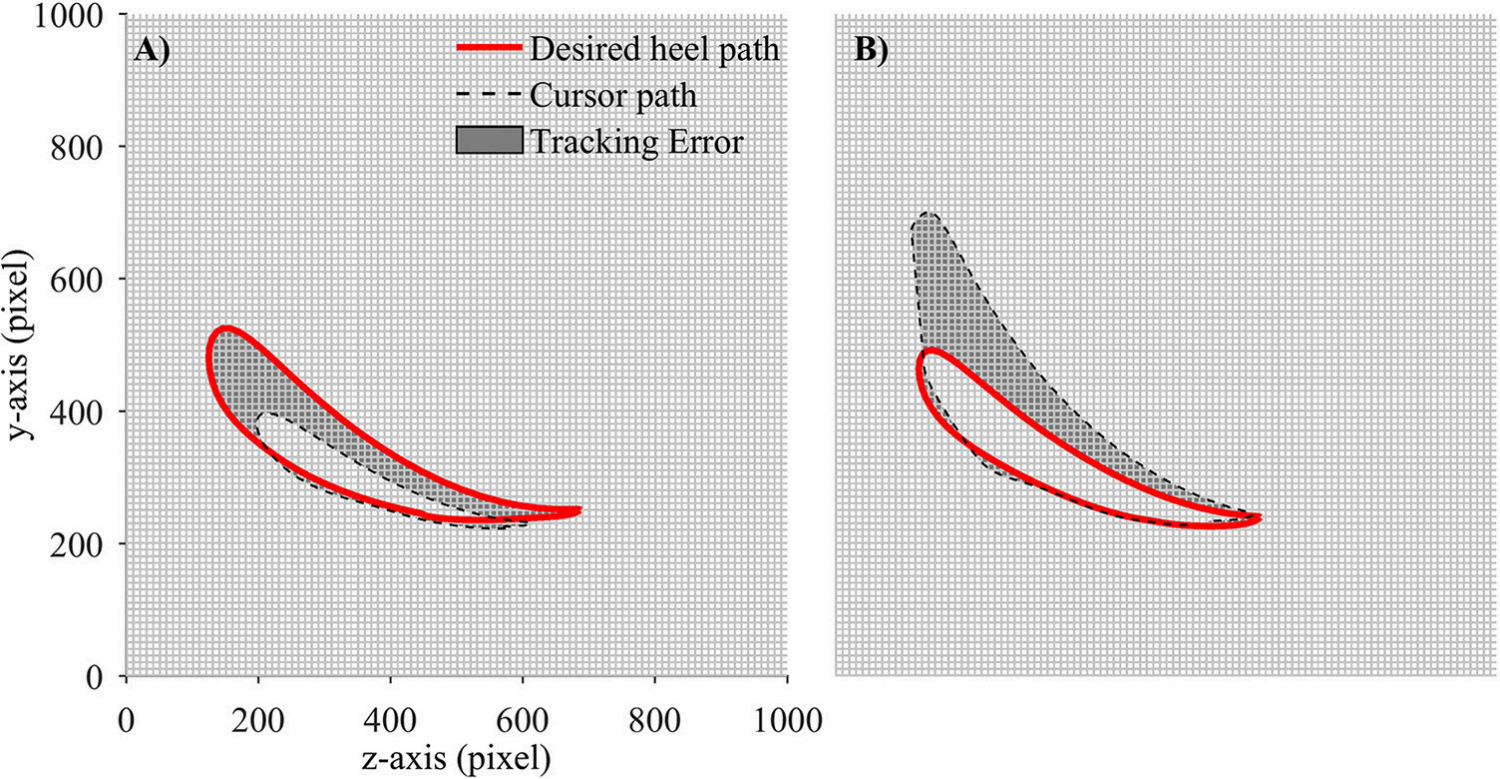
Computation of tracking error in each gait cycle. Desired heel path (solid red line) and cursor path (dashed black line) were placed on a 1,000 × 1,000 pixel image. Tracking error was defined as the number of pixels in gray area. Each small square block in the grid contains 100 pixels. **(A)** Tracking error is negative and **(B)** Tracking error is positive.

In order to evaluate the performance of participants in adapting to the visual kinematic perturbations, we defined tracking error and response time as performance measurements. Tracking error for each gait cycle was defined as the difference between the moving cursor path and the prescribed heel path (average of gait templates across gait cycles). A value closed to zero would signify that the moving cursor which represents the subjects' heel position followed the prescribed heel path profile correctly. We first converted the desired heel path and the moving cursor's path into regions and placed them on an image of 1,000 × 1,000 pixels. The tracking error was then computed by subtracting the area inside the moving cursor to the area inside the desired heel path (in pixels). Positive tracking error was defined when the area of the moving cursor was larger than the area of the desired heel path and vice versa. Figure 4 illustrates the computation of tracking error in one gait cycle.

**Figure 5:**
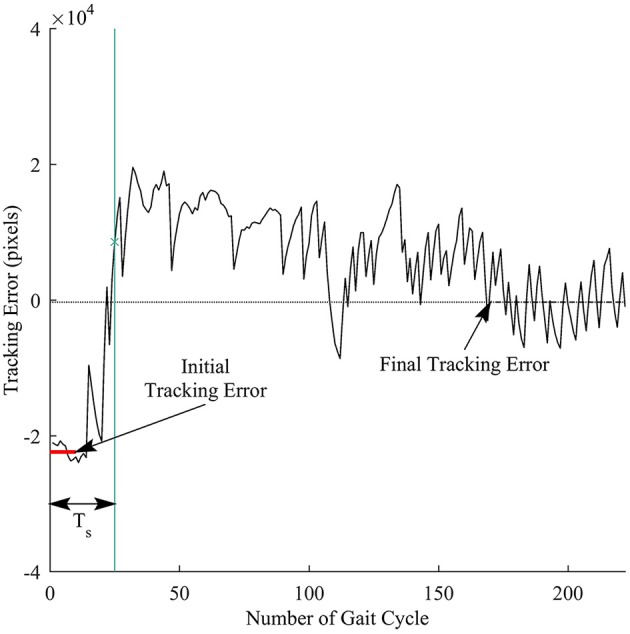
Illustration of response time definition for each trial. Thick red line represents the initial tracking error (at the beginning of perturbation), and dashed line represents final tracking error (at late exposure). T_s_ is the response time computed in the number of gait cycles. Vertical green line is time when a subject responded to the visual perturbations.

We defined response time to measure how fast a subject responded to the visual perturbations. Figure 5 illustrates the computation for the response time. First, the initial tracking error was computed as the average of tracking errors in the first 10 gait cycles before the subjects started responding to the perturbations, and the final tracking error was obtained by averaging tracking errors in the late exposure. We then applied cumulative sum (cusum) to track the deviations of each sample away from the initial tracking error. Cusum chart is especially useful in detecting if a signal has drifted beyond a pre-defined deviation above and below a target value. We identified the response time T_s_ by applying cusum with target value is the initial tracking error and the pre-defined deviation is the difference between the final and the initial tracking errors. The response time T_s_ was computed in the number of gait cycles.

### Assessment of the adaptations of lower limb joint angles across subjects

To analyze the adaptation of lower limb joint angles under the presence of the visual perturbations, we computed the ratio between the range of movement (ROM) in late-exposure (Trial 4) and the ROM in normal walking (without visual feedback). The ROM ratios were computed for all joints (hip, knee, and ankle) of the right leg.

### Assessment of gait symmetry during visual kinematic perturbations

The changes of gait symmetry under constraint of visual kinematic perturbations were measured in both temporal and spatial domains. Previous studies on locomotion adaptation using split-belt treadmill have demonstrated that the motor system may generate temporal motor outputs to minimize the difference in double support times when the subjects were walking under perturbations (Reisman et al., 2005; Malone et al., 2012). These studies also suggested that the temporal motor outputs from motor system approach a desired value defined as the normalized stance time difference. In this study, the gait symmetry index in the temporal domain is defined as follows:

(3) $$\begin{array}{l} S_{temporal}=\frac{ST_{l}-ST_{r}}{t_{r}+t_{l}} \end{array}$$

where *t*_*r*_ and *t*_*l*_ are right and left step times; *ST*_*r*_ and *ST*_*l*_ are stance times of right and left legs, respectively.

In the spatial domain, the differences between lower limb joint angles of the right and left legs were defined to characterize spatial asymmetry. The gait symmetry index in the spatial domain were defined as the difference between the range of motion (ROM) of the right and the left legs normalized by their sum:

(4) $$\begin{array}{l} S_{spatial}=\frac{ROM_{r\gamma }-ROM_{l\gamma }}{ROM_{r\gamma }+ROM_{l\gamma }} \end{array}$$

where γ is the angle between the vertical line (to the ground) and the line that connects right hip (greater trochanter) and heel (the posterior aspect of the calcaneus) positions.

Gait symmetry indices in both temporal and spatial domains were normalized by subtracting the mean of these values in the period when the subjects walked without the visual feedback on the screen. Gait symmetry values closed to zero indicate that a subject's gait pattern was highly symmetrical.

### Statistical analysis

Statistical analyses were performed to analyze the changes of tracking errors, which had non-normal distributions, across different phases in the 4 training trials: pre-exposure, exposure (from trial 1 to trial 4), and post-exposure. Kruskal-Wallis non-parametric statistical tests with Tukey-Kramer correction for *post-hoc* multiple comparison was applied to assess the changes of tracking errors and response time across all trials. This model was also applied to test the adaptation of the spatial and temporal gait symmetry across all trials. To compare the adaptation of lower limb joint angles, which had normal distributions, under the presence of perturbations, we used a within subject, repeated-measures one-way ANOVA test.

## Results

### Subjects adapted their gait in response to visual kinematic perturbations

**Figure 6:**
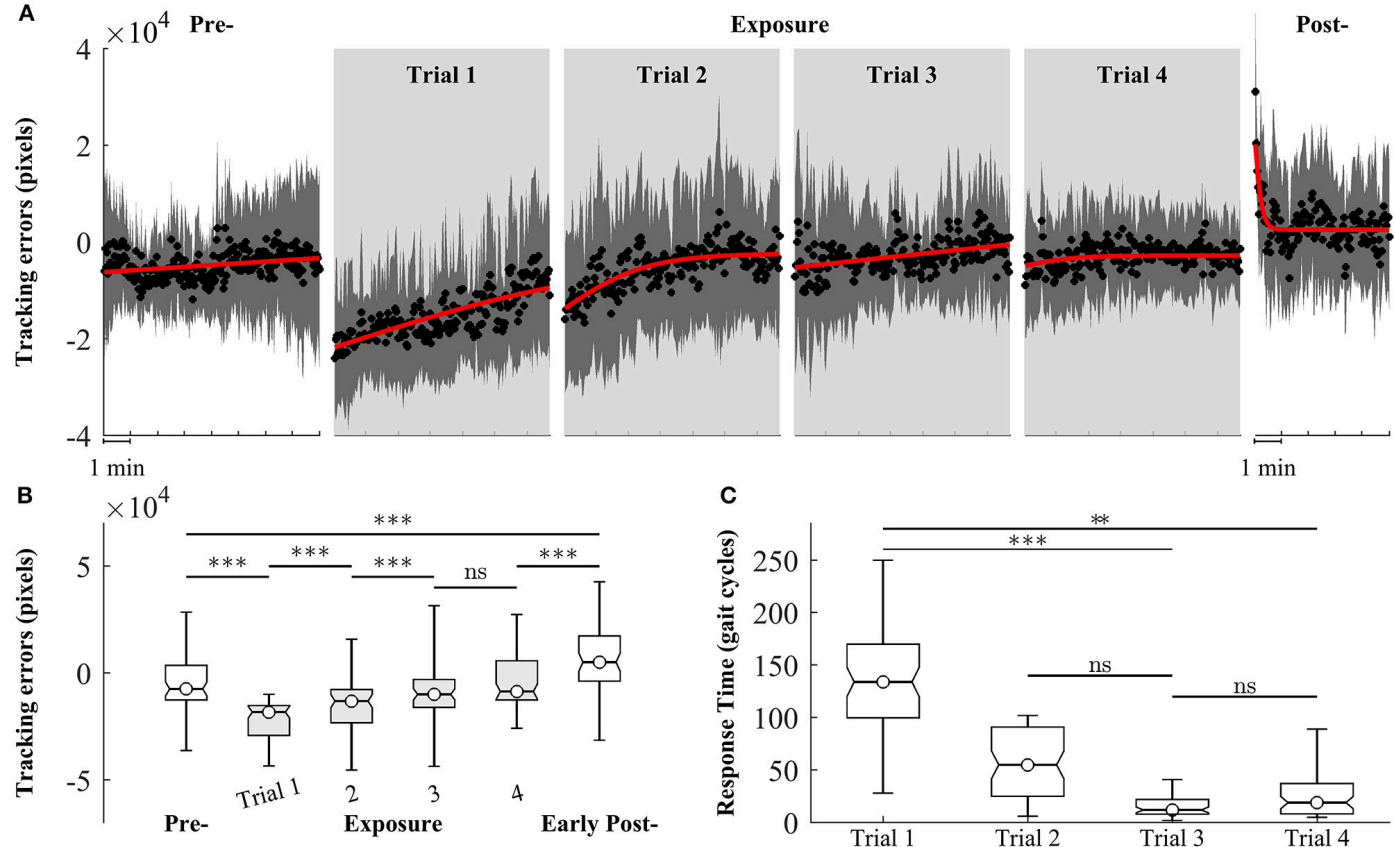
Group averages of tracking errors across 4 trials of training, gray areas represent early exposure period. **(A)** Black circles represent mean of tracking errors across subjects, and shaded areas indicate one standard deviation. Solid lines are fitting curves using a sigmoidal model. **(B,C)** Statistical analysis of tracking errors and response time using multiple comparison test with Tukey-Kramer adjustment, respectively. ^**^P < 0.01 and ^***^P < 0.001. ns, non-significant.

We observed changes in both tracking errors and response time across training trials. Figure 6A shows the group means of tracking errors in different phase of training: pre-, exposure, and post-. During pre-exposure, subjects walked normally on treadmill and the moving cursor was closed to the prescribed heel path. When visual kinematic perturbations were introduced (lower limb joint angles were scaled down by 0.7), the moving cursor departed from the prescribed heel path and subjects started adapting their gait to the perturbation. The tracking errors in the early exposures gradually decreased across trials (from Trial 1 to Trial 4) as subjects were adapting to the visual perturbation. When subjects resolved the perturbation problem, the moving cursor were closed the prescribed heel path profiles again. In the post-exposure phase, the visual kinematic perturbation was removed. In this phase, the moving cursor departed from the prescribed heel path profiles again but in the opposite direction (positive tracking errors), reflecting after-effects of gait adaptation. By the end of this trial, the tracking errors returned to pre-exposure levels.

Figure 6B shows the results of statistical analysis for tracking errors. Significant increases in tracking errors [block means (×10^4^ pixels): pre = −0.47 ± 1.21; early exposure (the first 30 gait cycles in Trial 1) = −2.07 ± 1.25, *P* < 0.001] were found immediately after the introduction of visual kinematic perturbations. As expected, the tracking errors gradually decreased with practice from Trial 1 to Trial 3 and reached a plateau in Trial 4 [block means for early-exposure (×10^4^ pixels): Trial 1 = −2.07 ± 1.25; Trial 2 = −0.54 ± 1.57; Trial 3 = −0.28 ± 1.26, and Trial 4 = −0.29 ± 0.97]. There were significant after-effects for tracking errors in the early post-exposure phase (the first 1 min) when the visual perturbation was removed [group means (×10^4^ pixels): pre = −0.47 ± 1.21; early post = 0.68 ± 1.54, *P* < 0.001]. Figure 6C shows the results of response time during exposure periods across 4 trials. When the visual perturbation was introduced in the first trial, the mean and one standard deviation of the response time across all subjects was 148.5 ± 69.1 gait cycles. These value substantially decreased in later trials except for Trial 4 (Trial 2: 56.2 ± 36.0; Trial 3: 19.3 ± 20.7; Trial 4: 35.7 ± 36.7). Although the response time in the trial 4 is higher than in the trial 3, the difference is non-significant (*p*-value > 0.05).

### Strategies of gait adaptation varied across subjects

**Figure 7:**
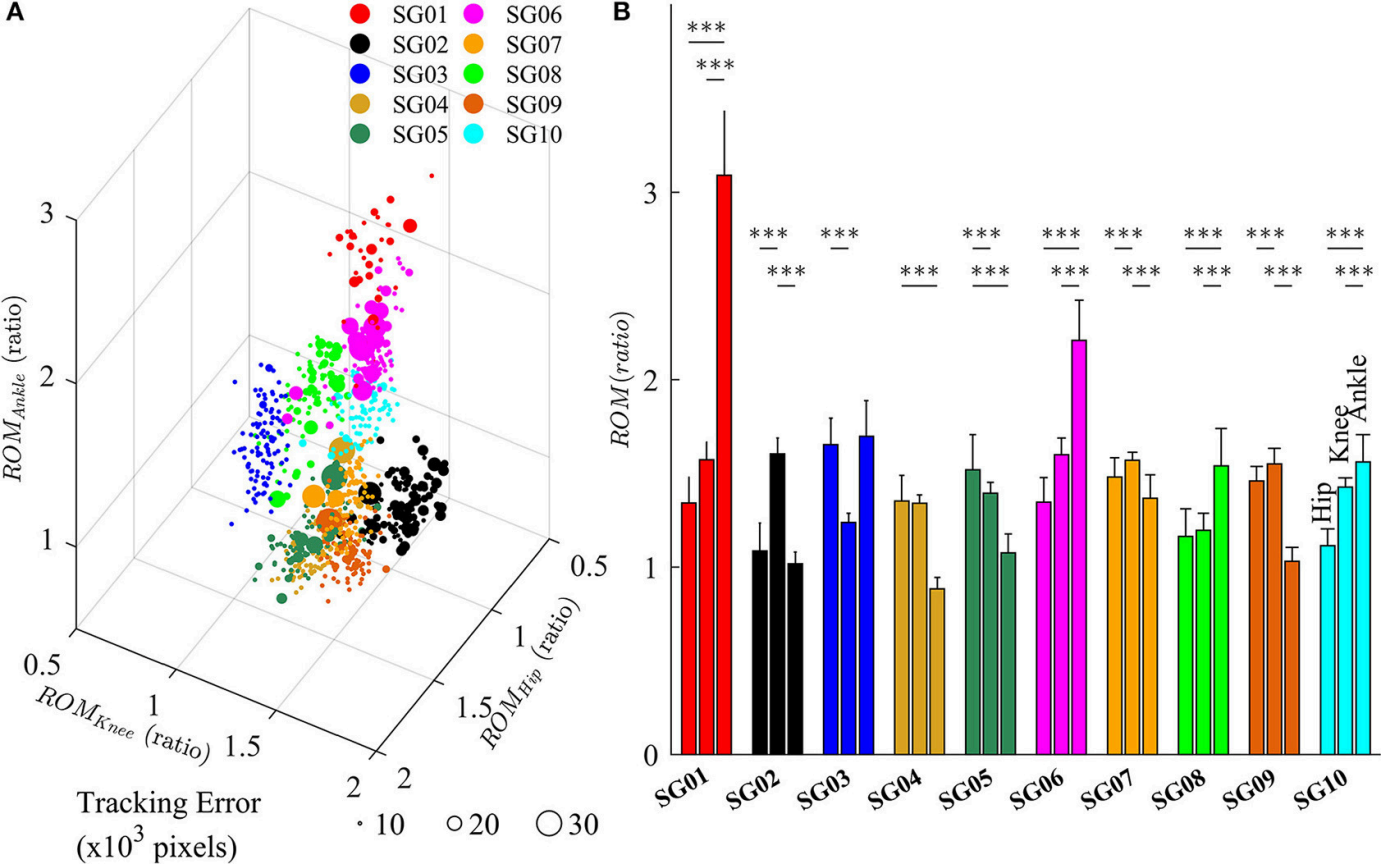
**(A)** Variations of lower limb joint angles (hip, knee, and ankle) in the right leg for 10 subjects in late-exposure (Trial 4). The size of the symbols represents the tracking error. **(B)** ROM ratio is the proportion of range of motion in late-exposure compared to pre-exposure. *Post-hoc* analyses using multiple comparison with Tukey-Kramer adjustment. ^***^P < 0.001.

Figure 7 depicts the changes in lower limb joint angles during late-exposure (Trial 4) when subjects adapted to the visual kinematic perturbations. Results from *post-hoc* analysis show that subjects varied joint angles differently when adapting to the perturbation. For example, SG01 had more changes in ankle joint angles (Hip: 1.35 ± 0.14; Knee: 1.57 ± 0.10; Ankle: 3.11 ± 0.34, *P* < 0.001), while SG02 had more changes in knee joint angles (Hip: 1.09 ± 0.15; Knee: 1.61 ± 0.09; Ankle: 1.02 ± 0.06, *P* < 0.001). Figure 7A shows that different strategies of gait adaptation affect tracking errors in the late-exposure. Specifically, the tracking errors of subjects SG01: 12.61 ± 6.95, SG06: 11.06 ± 6.25, and SG08: 10.46 ± 2.80 were significantly higher than the other subjects: 8.67 ± 4.45 (these values are in ×10^3^ pixels). Figure 7B reveals that SG01, SG06, and SG08 adapted more on the ankle joint angles instead of the hip and knee joints. This gait adaptation strategy resulted in larger tracking errors because the changes in the ankle joint angles have lesser effect on the variations of heel position as compared to the changes in the hip and knee joint angles (Equation 2).

### Gait symmetry in the spatial and temporal domains during visual kinematic perturbations

**Figure 8:**
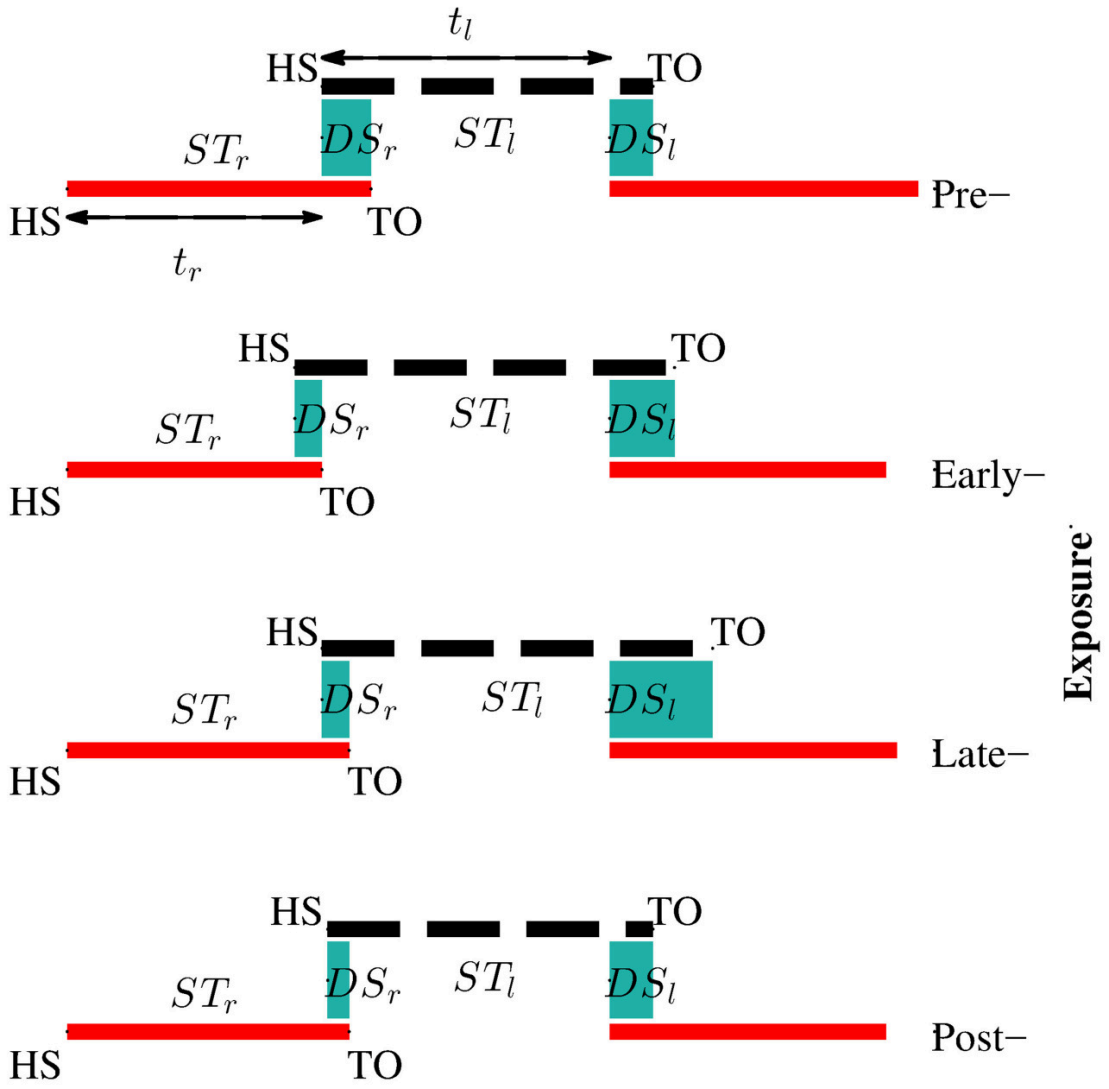
Adaptation of temporal parameters during visual kinematic perturbations for subject SG04. The solid red line and dashed black line represent stance periods of the right and left leg, respectively. Double support periods are represented by the shaded sea green area. During the pre-exposure phase, the subject walked normally and the right and left double support times were equal. Temporal asymmetry increased in the exposure phases and sustained in post-exposure.

Figure 8 illustrates the changes of gait symmetry in the temporal domain to visual kinematic perturbations. Theoretically, the right and left double support limb periods are equal during normal walking conditions (pre-exposure). When visual kinematic perturbations were introduced and subjects started adapting their gait to the perturbation, the double support periods are different and the gait asymmetry in temporal domain increase. In the post-exposure phase, visual kinematic perturbations were removed and double support periods gradually return to symmetric step times.

**Figure 9:**
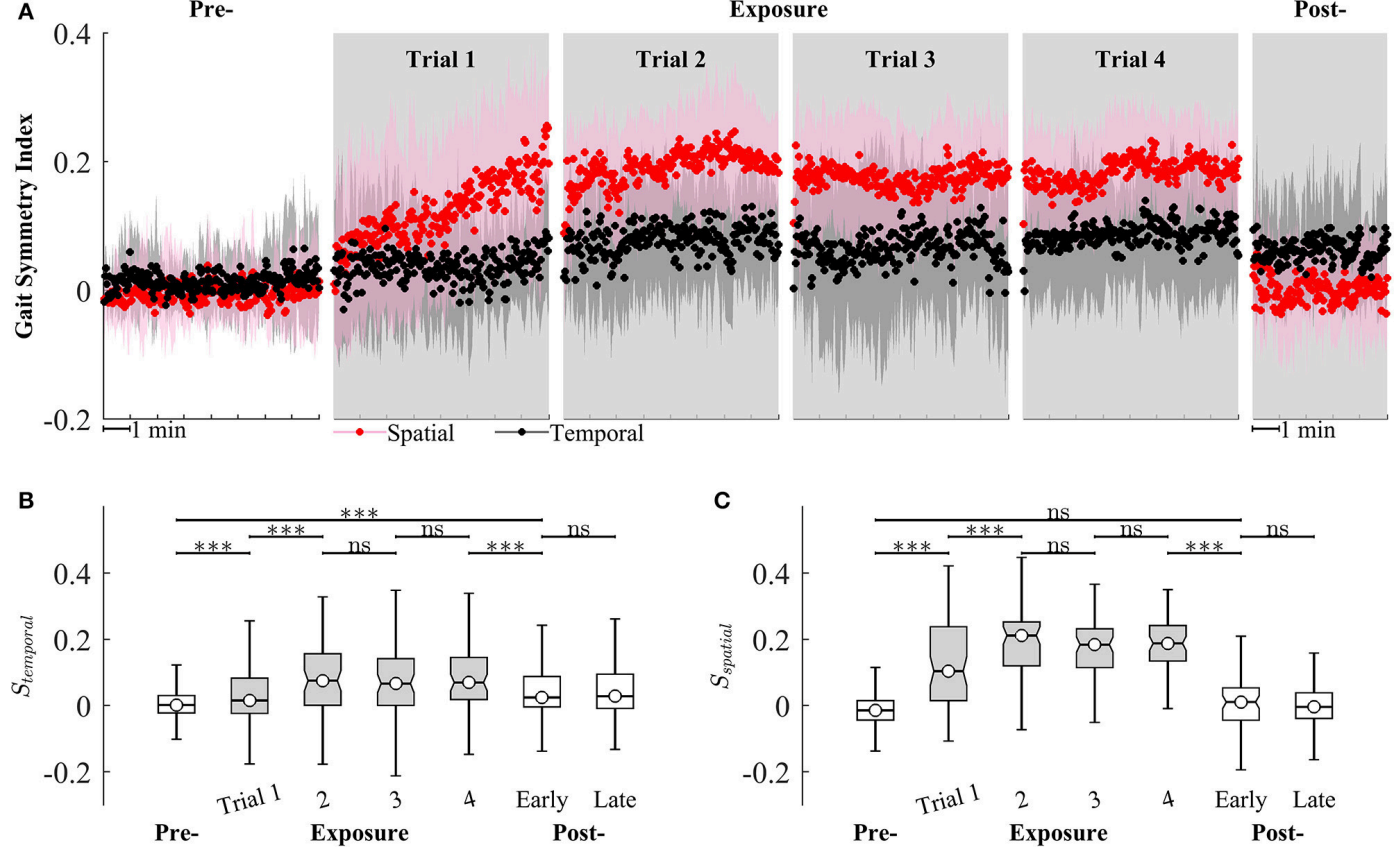
**(A)** Group averages of gait symmetry indices across 4 trials of training. Black and red circles represent mean values of temporal and spatial gait symmetry indices, respectively. Shaded areas indicate one standard deviation. **(B,C)** Statistical analysis for gait symmetry index in the temporal (*S*_*temporal*_), and spatial (*S*_*spatial*_) domain using multiple comparison with Tukey-Kramer adjustment. ^***^P < 0.001, *ns*, non-significant. Box plots in gray represent exposure period.

We observed that *S*_*spatial*_ and *S*_*temporal*_ can characterize the adaptation of gait symmetry in the spatial and temporal domains, respectively. For example, results from Figure 9 show that *S*_*spatial*_ and *S*_*temporal*_ significantly increased between the early (Trial 1) and late exposure phase (Trial 4), indicating the adaptation of motor outputs for gait symmetry (*S*_*spatial*_ − Trial 1: 0.09 ± 0.09, Trial 4: 0.19 ± 0.06, *P* < 0.001; *S*_*temporal*_ − Trial 1: 0.05 ± 0.07, Trial 4: 0.08 ± 0.08, *P* < 0.001). Moreover, there was significant difference between pre- and post-exposure (*S*_*temporal*_ − Pre: 0.02 ± 0.07, Post-Exposure: 0.06 ± 0.09, *P* < 0.001), indicating that there were significant storages of new gait symmetry indices in the temporal domains.

Figure 9 also illustrates that the gait symmetry in the temporal domain is less sensitive to the visual kinematic perturbations and its after-effects are larger than those in the spatial domain. For example, when the perturbations were introduced, *S*_*spatial*_ increased from −0.002 ± 0.07 (Pre-) to 0.12 ± 0.14 (Exposure, trial 1) while *S*_*temporal*_ increased from 0.02 ± 0.07 to 0.05 ± 0.07. When the perturbations were removed, *S*_*spatial*_ decreased from 0.18 ± 0.08 (Exposure, trial 4) to 0.006 ± 0.09 (Post-) while *S*_*temporal*_ decreased from 0.08 ± 0.08 to 0.06 ± 0.09. Moreover, gait symmetry index in the spatial domain approached a new-steady state faster than in the temporal domain. For example, *S*_*spatial*_ reached a new steady state on Trial 2 (*S*_*spatial*_ was not significantly different from Trial 2 to Trial 4) while *S*_*temporal*_ reached a new steady-state on Trial 4. The perturbation resulted in a stronger after-effect in the temporal domain as compared to the spatial domain. The temporal gait symmetry was significantly different between the pre- and post-exposure phase (*P* < 0.001) and it lasted for more than 5 min. However, there is no significant difference in the spatial gait symmetry index between pre- and post-exposure phase. When the perturbation was removed, the spatial gait symmetry index returned to the pre-exposure condition in <1 min.

## Discussion

This study investigated gait adaptation of healthy individual in the presences of visual kinematic perturbation (i.e., a gain change) of gait during treadmill walking across several trials. The visual kinematic perturbations, which caused a mismatch between actual and prescribed heel path trajectories displayed on a screen, were introduced to investigate visual-motor adaptation of human locomotion. We observed that, with practice, the subjects could reduce the errors induced by the visual kinematic perturbations and showed faster response time in later trials (Trial 1 to Trial 3). Thus, by late exposure, subjects have found a solution for the visual kinematic perturbations problem and acquired, at least partially, the internal model of the visuomotor transformation. Moreover, the strategies of gait adaptation varied across subjects and affected steady-state tracking errors in late-exposure. Our results also showed that the temporal gait symmetry in the post-exposure phase was significantly different from the pre-exposure phase. This after-effect suggested that there were significant storages of new temporal gait symmetry index.

Introduction of the kinematic gait perturbation showed an initial rise of tracking errors in early-exposure, the reduction of tracking errors in exposure, and the after-effect in post-exposure (Figure 6). The presence of after-effects is an indication that motor adaptation has occurred (Shadmehr and Mussa-Ivaldi, 1994; Kagerer et al., 1997; Buch et al., 2003; Krakauer et al., 2005; Scheidt et al., 2005; Choi and Bastian, 2007; Reisman et al., 2009; Kim et al., 2010). Although the experimental setup in this study is simple, the results reflect the decreases of tracking errors across trials and the after-effects that characterize the motor adaptation process. We hypothesize that the training method used in this study could be a feasible intervention to potentially improve asymmetries in post-stroke gait rehabilitation. Previous studies have shown that after-effects of locomotor or visuomotor adaptation improve task performance in individuals post-stroke (Rossetti et al., 1998; Patton et al., 2006). Moreover, Reisman et al. demonstrated that the improvements of task performance following split-belt treadmill adaptation could transfer to real world tasks such as overground walking (Reisman et al., 2009).

Gait asymmetry is an important gait characteristic that may have a role in guiding the clinician's treatment decisions (Patterson et al., 2008, 2010). Gait asymmetry may be associated with negative effects on human locomotion such as decreases in gait efficiency, balance control, and gait speed (Jorgensen et al., 2000). For example, asymmetric step length results in decreased walking speed, and both decreased propulsive force and increased severity of the paretic limb (Bowden et al., 2006; Balasubramanian et al., 2007; Jonkers et al., 2009). Asymmetric double support time is related to decreased gait speed (Olney et al., 1994). Asymmetric walking gait patterns are commonly found in individuals post-stroke. However, some individuals post-stroke have asymmetry in only one domain. For example, they only have either step length asymmetry (spatial) or double support time asymmetry (temporal). Therefore, it could be more efficient to target therapeutic rehabilitation to only the domain (spatial or temporal) of gait symmetry in which the post-stroke persons experience difficulty. Separate control of the temporal and spatial control of human locomotion has been demonstrated in studies of locomotor adaptation to split-belt treadmill training (Reisman et al., 2009; Malone et al., 2012). However, interventions that target only one domain of gait asymmetry have not been fully developed. In this study, multi-trial locomotor training using visual kinematic perturbations was implemented and the subjects adapted to the perturbations by modifying their gait patterns. Once adapted, the subjects could not retrieve their normal gait pattern immediately when the perturbations were removed. Instead, they had to “de-adapt” their gait patterns back to original state. This after-effect was a strong clue that motor adaptation has occurred and a new representation of visuomotor mapping has been learned. Results in Figure 9 show the after-effects of gait symmetry indices in the temporal domains in early post-exposure phase, indicating that there were significant storages of new temporal gait symmetry index. However, after-effects were not observed for the spatial gait symmetry index. These findings have implications for a possible intervention to improve gait symmetry in the temporal domain for individuals post-stroke and the setup in this study could be beneficial to gait rehabilitation.

Our results suggest differential temporal and spatial symmetry effects to the kinematic perturbations. For example, we observed after-effects of the temporal gait symmetry index after the presence of kinematic perturbations, indicating its adaptation. However, the after-effect or adaptation of the spatial gait symmetry was missing. These results support previous findings suggesting that the adaptation of spatial and temporal gait symmetry is dissociable (Malone et al., 2012). The tracking task in this study was mainly spatial because the subjects followed the desired heel path without considering the time to reach a specific point. Interestingly, the adaptation occurred nevertheless in the temporal gait symmetry. This could have the implication that the subjects unconsciously adapted temporal gait symmetry under the visual kinematic perturbations. This findings are aligned with previous studies suggesting that subjects may not be able to consciously prevent adapting temporal motor output under split-belt conditions (Malone et al., 2012), and that temporal gait symmetry is harder to influence with conscious efforts (Malone and Bastian, 2010). Additionally, our findings support the hypothesis that the temporal control of human gait is more automatic and depends more heavily on subcortical circuits. Vasudevan et al. (2011) also showed that temporal adaptation was found to be fully developed earlier than spatial adaptation (by 3 year old and until adolescence, respectively).

The feasibility of this intervention to improve gait asymmetry might also be extended to target only specific joints that may be affected more than others. Our results demonstrated that subjects use multiple solutions, i.e., multiple ways for subjects to alter their joint movements, to adapt to the visual kinematic perturbations (Figure 7). For example, while the subject SG01 preferred to adapt the ankle joint angles, the subject SG02 favored to adapt the knee joint angles instead. Figure 7 illustrates that different strategies of gait adaptation affect tracking errors in late-exposure. Moreover, Figure 7B reveals that subjects who adapted more on the ankle joint angle instead of the hip and knee joints (SG01, SG06, and SG08) showed larger steady-state tracking errors in the late-exposure phase. This gait adaptation strategy resulted in higher tracking errors because the changes in the ankle joint angles have lesser effect on the variations of the heel positions as compared to the changes in the hip and knee joint angles (Equation 2). Overall, these findings are supported by the motor equivalence problem (Bernstein, 1967), which characterizes the kinematic redundancy of motor control systems: in this case, using three degrees of freedom (hip, knee, and ankle joints) to control the heel position in a two-dimensional space. Therefore, even though there are multiple solutions (due to redundant degrees of freedom) to accomplish a motor task, we can indirectly influence the motor system to favor one solution by controlling the rules that generate visual kinematic perturbations (i.e., using different scaling factors for each joint angle), which can allow for personalized gait therapy based on the current state of the patient.

Findings of this study are currently limited to healthy individuals. Future investigations should consider implementing the framework of visual kinematic perturbations for gait rehabilitation in individuals post-stroke. In this regard, the motor task is expected to be more challenging for persons post-stroke. The number of trials required to achieve significant performance improvement may also increase. It will also be important to examine the changes in the representations of gait at cortical level during the visual kinematic perturbations. The understanding of neural mechanisms of human gait adaptation is important for designing an effective training paradigm of gait rehabilitation in a top-down approach. Noninvasive electroencephalography (EEG) studies could be designed to investigate the underlying patterns of neural activity during the visual kinematic perturbations.

## Author contributions

TPL contributed to task design, developing software for data collection, acquisition of the data, interpretation for the work, and manuscript draft. YH contributed to developing software for data collection, acquisition of the data, and literature review. SN, KN, and JG contributed to experimental design, data acquisition, and revising the draft. SB contributed in developing software for data collection and manuscript review. JC conceived the experiment, edited the manuscript and approved the final version of the paper.

### Conflict of interest statement

The authors declare that the research was conducted in the absence of any commercial or financial relationships that could be construed as a potential conflict of interest.

## References

[B1] Alexander M. S. Flodin B. W. Marigold D. S. (2013). Changes in task parameters during walking prism adaptation influence the subsequent generalization pattern. J. Neurophysiol. 109, 2495–2504. 10.1152/jn.00810.2012 23446691

[B2] Antonsson E. K. Mann R. W. (1985). The frequency content of gait. J. Biomech. 18, 39–47. 10.1016/0021-9290(85)90043-0 3980487

[B3] Balasubramanian C. K. Bowden M. G. Neptune R. R. Kautz S. A. (2007). Relationship between step length asymmetry and walking performance in subjects with chronic hemiparesis. Arch. Phys. Med. Rehabil. 88, 43–49. 10.1016/j.apmr.2006.10.004 17207674

[B4] Bastian A. J. (2008). Understanding sensorimotor adaptation and learning for rehabilitation. *Curr. Opin*. Neurol. 21:628 10.1097/WCO.0b013e328315a293 PMC2954436 18989103

[B5] Bernstein N. A. (1967). The Co-ordination and Regulation of Movements. Oxford: Pergamon Press.

[B6] Bowden M. G. Balasubramanian C. K. Neptune R. R. Kautz S. A. (2006). Anterior-posterior ground reaction forces as a measure of paretic leg contribution in hemiparetic walking Stroke 37, 872–876. 10.1161/01.STR.0000204063.75779.8d 16456121

[B7] Buch E. R. Young S. Contreras-Vidal J. L. (2003). Visuomotor adaptation in normal aging. Learn. Mem. 10, 55–63. 10.1101/lm.50303 12551964 PMC196655

[B8] Cheng S. Sabes P. N. (2007). Calibration of visually guided reaching is driven by error-corrective learning and internal dynamics. J. Neurophysiol. 97, 3057–3069. 10.1152/jn.00897.2006 17202230 PMC2536620

[B9] Choi J. T. Bastian A. J. (2007). Adaptation reveals independent control networks for human walking. Nat. Neurosci. 10, 1055–1062. 10.1038/nn1930 17603479

[B10] Contreras-Vidal J. L. Kerick S. E. (2004). Independent component analysis of dynamic brain responses during visuomotor adaptation Neuroimage 21, 936–945. 10.1016/j.neuroimage.2003.10.037 15006660

[B11] Emken J. L. Benitez R. Sideris A. Bobrow J. E. Reinkensmeyer D. J. (2007). Motor adaptation as a greedy optimization of error and effort. *J*. Neurophysiol. 97, 3997–4006. 10.1152/jn.01095.2006 17392418

[B12] Finley J. M. Statton M. A. Bastian A. J. (2014). A novel optic flow pattern speeds split-belt locomotor adaptation *J*. Neurophysiol. 111, 969–976. 10.1152/jn.00513.2013 24335220 PMC3949224

[B13] Helm E. E. Reisman D. S. (2015). The split-belt walking paradigm: exploring motor learning and spatiotemporal asymmetry poststroke. Phys. Med. Rehabil. Clin. N. Am. 26, 703–713. 10.1016/j.pmr.2015.06.010 26522907 PMC4631066

[B14] Hussain S. J. Hanson A. S. Tseng S. C. Morton S. M. (2013). A locomotor adaptation including explicit knowledge and removal of postadaptation errors induces complete 24-hour retention. *J*. Neurophysiol. 110, 916–925. 10.1152/jn.00770.2012 PMC3742972 23741038

[B15] Imamizu H. Uno Y. Kawato M. (1998). Adaptive internal model of intrinsic kinematics involved in learning an aiming task. *J*. Exp. Psychol. Hum. Percept. Perform. 24:812 10.1037/0096-1523.24.3.812 9627418

[B16] Izawa J. Rane T. Donchin O. Shadmehr R. (2008). Motor adaptation as a process of reoptimization. *J*. Neurosci. 28, 2883–2891. 10.1523/JNEUROSCI.5359-07.2008 PMC2752329 18337419

[B17] Izawa J. Shadmehr R. (2011). Learning from sensory and reward prediction errors during motor adaptation. PLoS Comput. Biol. 7:e1002012. 10.1371/journal.pcbi.1002012 21423711 PMC3053313

[B18] Jonkers I. Delp S. Patten C. (2009). Capacity to increase walking speed is limited by impaired hip and ankle power generation in lower functioning persons post-stroke. Gait Posture 29, 129–137. 10.1016/j.gaitpost.2008.07.010 18789692 PMC2929166

[B19] Jorgensen L. Crabtree N. Reeve J. Jacobsen B. (2000). Ambulatory level and asymmetrical weight bearing after stroke affects bone loss in the upper and lower part of the femoral neck differently: bone adaptation after decreased mechanical loading. Bone 27, 701–707. 10.1016/S8756-3282(00)00374-4 11062359

[B20] Kagerer F. A. Contreras-Vidal J. L. Stelmach G. E. (1997). Adaptation to gradual as compared with sudden visuo-motor distortions. Exp. Brain Res. 115, 557–561. 10.1007/PL00005727 9262212

[B21] Kim S. H. Banala S. K. Brackbill E. A. Agrawal S. K. Krishnamoorthy V. Scholz J. P. (2010). Robot-assisted modifications of gait in healthy individuals. Exp. Brain Res. 202, 809–824. 10.1007/s00221-010-2187-5 20186402

[B22] Krakauer J. W. Ghez C. Ghilardi M. F. (2005). Adaptation to visuomotor transformations: consolidation, interference, and forgetting. J. Neurosci. 25, 473–478. 10.1523/JNEUROSCI.4218-04.2005 15647491 PMC6725486

[B23] Long A. W. Roemmich R. T. Bastian A. J. (2016). Blocking trial-by-trial error correction does not interfere with motor learning in human walking. J. Neurophysiol. 115, 2341–2348. 10.1152/jn.00941.2015 26912598 PMC4922458

[B24] Luu T. P. He Y. Brown S. Nakagame S. Contreras-Vidal J. L. (2016). Gait adaptation to visual kinematic perturbations using a real-time closed-loop brain–computer interface to a virtual reality avatar. J. Neural Eng. 13:036006. 10.1088/1741-2560/13/3/036006 27064824 PMC5726869

[B25] Luu T. P. He Y. Brown S. Nakagome S. Contreras-Vidal J. L. (2015). A closed-loop brain computer interface to a virtual reality avatar: gait adaptation to visual kinematic perturbations, in Virtual Rehabilitation Proceedings (ICVR), 2015 International Conference on (Valencia), 30–37. 10.1109/ICVR.2015.7358598 PMC5048680 27713915

[B26] Luu T. P. Low K. H. Qu X. Lim H. B. Hoon K. H. (2014). An individual-specific gait pattern prediction model based on generalized regression neural networks. Gait Posture 39, 443–448. 10.1016/j.gaitpost.2013.08.028 24071020

[B27] Malfait N. Shiller D. M. Ostry D. J. (2002). Transfer of motor learning across arm configurations. J. Neurosci. 22, 9656–9660. Available online at: http://www.jneurosci.org/content/22/22/9656.long 12427820 10.1523/JNEUROSCI.22-22-09656.2002 PMC6757833

[B28] Malone L. A. Bastian A. J. (2010). Thinking about walking: effects of conscious correction versus distraction on locomotor adaptation. J. Neurophysiol. 103, 1954–1962. 10.1152/jn.00832.2009 20147417 PMC2853281

[B29] Malone L. A. Bastian A. J. Torres-Oviedo G. (2012). How does the motor system correct for errors in time and space during locomotor adaptation? J. Neurophysiol. 108, 672–683. 10.1152/jn.00391.2011 22514294 PMC4073916

[B30] Malone L. A. Vasudevan E. V. Bastian A. J. (2011). Motor adaptation training for faster relearning. *J*. Neurosci. 31, 15136–15143. 10.1523/JNEUROSCI.1367-11.2011 PMC3209529 22016547

[B31] Morton S. M. Bastian A. J. (2004). Prism adaptation during walking generalizes to reaching and requires the cerebellum. *J*. Neurophysiol. 92, 2497–2509. 10.1152/jn.00129.2004 15190088

[B32] Nemanich S. T. Earhart G. M. (2015). Prism adaptation in Parkinson disease: comparing reaching to walking and freezers to non-freezers. Exp. Brain Res. 233, 2301–2310. 10.1007/s00221-015-4299-4 25976516 PMC4513667

[B33] Olney S. J. Griffin M. P. McBride I. D. (1994). Temporal, kinematic, and kinetic variables related to gait speed in subjects with hemiplegia: a regression approach. Phys. Ther. 74, 872–885. 10.1093/ptj/74.9.872 8066114

[B34] Patterson K. K. Parafianowicz I. Danells C. J. Closson V. Verrier M. C. Staines W. R.. (2008). Gait asymmetry in community-ambulating stroke survivors. Arch. Phys. Med. Rehabil. 89, 304–310. 10.1016/j.apmr.2007.08.142 18226655

[B35] Patterson K. K. Gage W. H. Brooks D. Black S. E. McIlroy W. E. (2010). Evaluation of gait symmetry after stroke: a comparison of current methods and recommendations for standardization. Gait Posture 31, 241–246. 10.1016/j.gaitpost.2009.10.014 19932621

[B36] Patton J. L. Stoykov M. E. Kovic M. Mussa-Ivaldi F. A. (2006). Evaluation of robotic training forces that either enhance or reduce error in chronic hemiparetic stroke survivors. Exp. Brain Res. 168, 368–383. 10.1007/s00221-005-0097-8 16249912

[B37] Reisman D. S. Block H. J. Bastian A. J. (2005). Interlimb coordination during locomotion: what can be adapted and stored? J. Neurophysiol. 94, 2403–2415. 10.1152/jn.00089.2005 15958603

[B38] Reisman D. S. Wityk R. Silver K. Bastian A. J. (2007). Locomotor adaptation on a split-belt treadmill can improve walking symmetry post-stroke. Brain 130, 1861–1872. 10.1093/brain/awm035 17405765 PMC2977955

[B39] Reisman D. S. Wityk R. Silver K. Bastian A. J. (2009). Split-belt treadmill adaptation transfers to overground walking in persons poststroke. Neurorehabil. Neural Repair 23, 735–744. 10.1177/1545968309332880 19307434 PMC2811047

[B40] Rossetti Y. Rode G. Pisella L. Farné A. Li L. Boisson D.. (1998). Prism adaptation to a rightward optical deviation rehabilitates left hemispatial neglect Nature 395, 166–169. 10.1038/25988 9744273

[B41] Scheidt R. A. Conditt M. A. Secco E. L. Mussa-Ivaldi F. A. (2005). Interaction of visual and proprioceptive feedback during adaptation of human reaching movements. *J*. Neurophysiol. 93, 3200–3213. 10.1152/jn.00947.2004 15659526

[B42] Scheidt R. A. Dingwell J. B. Mussa-Ivaldi F. A. (2001). Learning to move amid uncertainty. J. Neurophysiol. 86, 971–985. Available online at: http://jn.physiology.org/content/86/2/971.short 11495965 10.1152/jn.2001.86.2.971

[B43] Shadmehr R. Mussa-Ivaldi F. A. (1994). Adaptive representation of dynamics during learning of a motor task. *J*. Neurosci. 14, 3208–3224. 10.1523/JNEUROSCI.14-05-03208.1994 PMC6577492 8182467

[B44] Statton M. A. Toliver A. Bastian A. J. (2016). A dual-learning paradigm can simultaneously train multiple characteristics of walking. *J*. Neurophysiol. 115, 2692–2700. 10.1152/jn.00090.2016 PMC4922483 26961100

[B45] Torres-Oviedo G. Bastian A. J. (2010). Seeing is believing: effects of visual contextual cues on learning and transfer of locomotor adaptation. *J*. Neurosci. 30, 17015–17022. 10.1523/JNEUROSCI.4205-10.2010 PMC3025449 21159971

[B46] Torres-Oviedo G. Vasudevan E. Malone L. Bastian A. J. (2011). Locomotor adaptation. Prog. Brain Res. 191, 65–74. 10.1016/B978-0-444-53752-2.00013-8 21741544 PMC3738197

[B47] Vasudevan E. V. Torres-Oviedo G. Morton S. M. Yang J. F. Bastian A. J. (2011). Younger is not always better: development of locomotor adaptation from childhood to adulthood. *J*. Neurosci. 31, 3055–3065. 10.1523/JNEUROSCI.5781-10.2011 21414926 PMC3084584

[B48] Wei K. Kording K. (2009). Relevance of error: what drives motor adaptation? J. Neurophysiol. 101, 655–664. 10.1152/jn.90545.2008 19019979 PMC2657056

[B49] Winter D. A. (1992). Foot trajectory in human gait: a precise and multifactorial motor control task. Phys. Ther. 72, 45–53. 10.1093/ptj/72.1.45 1728048

