# Supplementary material for: Multi-Trial Gait Adaptation of Healthy Individuals during Visual Kinematic Perturbations
Source: Front Hum Neurosci. 2017 Jun 20;11:320. doi: 10.3389/fnhum.2017.00320 (PMC5476704; doi:10.3389/fnhum.2017.00320)
Supplement: Supplementary file 1 [file Presentation1.pdf]

Supplementary Material

Multi-trial gait adaptation of healthy individuals during visual kinematic perturbations

Trieu Phat Luu, Yongtian He, Sho Nakagome, Kevin Nathan, Samuel Brown, Jeffrey Gorges, and Jose L. Contreras-Vidal

Noninvasive Brain-Machine Interface System Laboratory,

Dept. of Electrical and Computer Engineering,

University of Houston, Houston, TX 77004, USA.

Correspondence:

Trieu Phat Luu. Email: ptluu2@central.uh.edu

Conflict of Interest: The authors have no conflicts of interest.

Acknowledgements

This research was supported by award R01NS075889 from the National Institute of Neurological Disorders And Stroke (NINDS). The content is solely the responsibility of the authors and does not necessarily represent the official views of the NINDS or the National Institutes of Health (NIH).

Supplementary Figure 1 illustrates the procedure of the goniometer setup for the right leg. The final locations of the goniometer sensors at the hip, knee, and ankle joints after the setup is shown in Figure 1A. At the beginning of each session (day), we attached all six goniometer sensors to custom 3D printed tools. The design of these tools was based on the dimensions of the goniometer sensors and anthropometric data of healthy adult individuals (i.e., average distance from the lateral malleolus of the fibula to the ground at standing position). Prior to placing the goniometer sensors onto the subjects' left and right legs, we visually identified their hip and knee joint locations. To locate the hip joint position, we instructed the subjects to stand straight up and gradually lift one leg until the angle between their thigh and trunk was 90 degrees. The joint center was identified by palpation, and the goniometer was attached after the subject returned to a standing position.

Figures 1B), 1C), and 1D) show the placement of goniometers at the hip, knee, and ankle joint, respectively. Medical tape was used to secure the sensors in place. Finally, we recorded the distance from the sensors' lower edges (i.e., hip and knee) to the ground, as illustrated in Figure 1E) and 1F). These values were used to improve the consistency of placing the goniometer sensors in the following days.

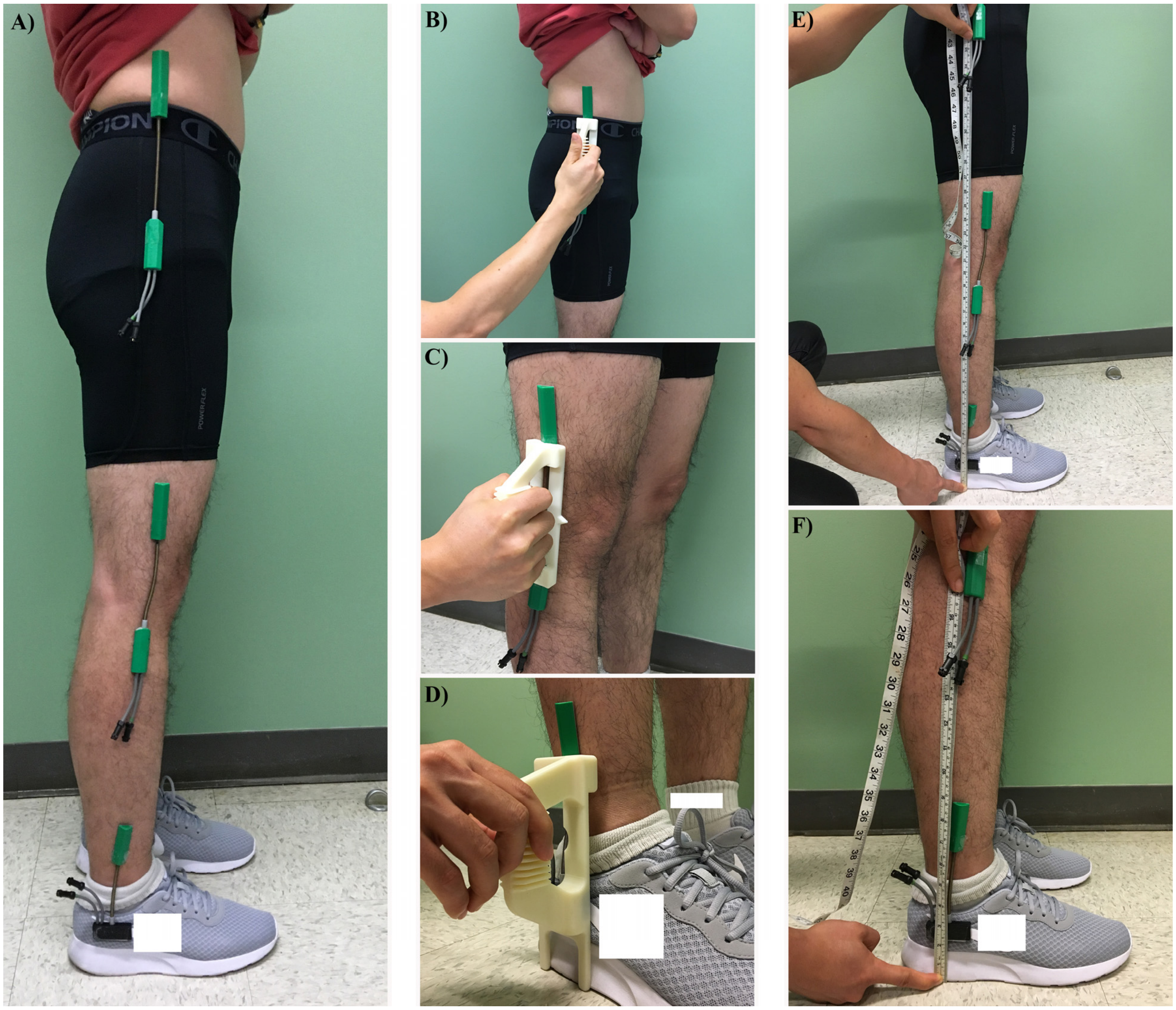

Supplementary Figure 1: Example of goniometer sensor setup in this study. A) Location of goniometer sensors to measure right hip, knee, and ankle joint angles. B) to D) Custom 3D printed tools were used to place goniometer sensors at hip, knee, and ankle joint, respectively. E) and F) Distance from lower edge of goniometer sensors (hip and knee, respectively) to the ground were recorded to improve consistency for the experimental setup in the following days.
